# From Emulsions to Films: The Role of Polysaccharide Matrices in Essential Oil Retention Within Active Packaging Films

**DOI:** 10.3390/foods14091501

**Published:** 2025-04-25

**Authors:** Elisa Othero Nahas, Guilherme F. Furtado, Melina S. Lopes, Eric Keven Silva

**Affiliations:** 1 Graduate Program in Chemical Engineering, Institute of Science and Technology, Federal University of Alfenas-Campus Poços de Caldas, Poços de Caldas 37715-400, MG, Brazil; elisa.othero@sou.unifal-mg.edu.br (E.O.N.); melina.savioli@unifal-mg.edu.br (M.S.L.); 2 Centro de Ciências da Natureza, Universidade Federal de São Carlos (UFSCar), Rod. Lauri Simõoes de Barros, km 12—SP 189, Buri 18290-000, SP, Brazil; gfurtado@ufscar.br; 3 Faculdade de Engenharia de Alimentos (FEA), Universidade Estadual de Campinas (UNICAMP), Rua Monteiro Lobato, 80, Campinas 13083-862, SP, Brazil

**Keywords:** casting technique, limonene, encapsulation efficiency, antimicrobial, O/W emulsions

## Abstract

Essential oil-loaded edible films have emerged as promising natural systems for active food packaging due to their antimicrobial and antioxidant potential. However, retaining volatile bioactives within hydrophilic matrices remains challenging. In this regard, this study compared the performance of carboxymethylcellulose (CMC), citrus peel pectin (CPP), and potato starch (PS) edible films as encapsulating systems of orange (*Citrus sinensis* L. Osbeck) essential oil using Tween 80 as surfactant and glycerol as a plasticizer. Film-forming emulsions were characterized regarding droplet size distribution and rheological behavior. Films were analyzed by scanning electron microscopy (SEM), Fourier-transform infrared spectroscopy (FTIR), X-ray diffraction (XRD), and thermogravimetric analysis (TGA). Limonene retention was quantitatively determined post-drying through gas chromatography. CMC-based films exhibited the highest retention (~65%), primarily due to their higher viscosity, which limited oil droplet mobility and volatilization. Despite presenting similar internal porosity, PS films showed significantly lower retention (~53%), attributed to larger droplet size and lower viscosity. CPP films, with the smallest droplets and intermediate viscosity, showed similar limonene retention to PS-based films, highlighting that high internal porosity may compromise encapsulation efficacy. The results emphasize that matrix viscosity and emulsion stability are critical determinants of essential oil retention. Although polysaccharide films, particularly CMC, are promising carriers, further structural and processing optimizations are required to enhance their encapsulation performance for commercial applications.

## 1. Introduction

Essential oils are defined as volatile, complex mixtures of aromatic compounds extracted from various plant parts, such as flowers, leaves, and roots. Characterized by their distinct fragrances and intricate chemical compositions, these oils primarily consist of terpenes and phenolic compounds, which contribute to their diverse biological activities. Consequently, essential oils find widespread applications in foods, aromatherapy, perfumery, cosmetics, and, increasingly, in pharmaceutical research due to their reported antimicrobial, antioxidant, and anti-inflammatory properties [1,2].

Over recent years, there has been a growing interest in incorporating essential oils into active edible films for food packaging applications [3,4,5]. These materials are thin layers of edible biopolymers, such as polysaccharides or proteins, that incorporate active compounds, such as essential oils, to enhance food preservation and safety. The incorporation of essential oils provides these films with antimicrobial and antioxidant properties, making them suitable for extending the shelf life of various food products, preventing spoilage, and potentially delivering health-promoting compounds [6,7].

Despite the growing number of research on the incorporation of essential oils into active edible films, a critical gap remains in the evaluation of these biopolymeric systems as efficient carriers for these volatile compounds. While studies often demonstrate the simple incorporation of essential oils, there is a relative scarcity of work assessing how much of the essential oil is effectively retained within the film structure after the drying process of the film-forming emulsion. This contrasts with the microencapsulation of essential oils via techniques like spray-drying, where retention or encapsulation efficiency is a central focus of investigation [8,9]. The challenge lies in the fact that film-forming emulsions are subjected to drying methods such as casting or extrusion, raising the crucial question of the actual amount of essential oil that remains entrapped within the final film matrix. This under-explored aspect in the literature warrants significant attention to fully understand the potential and limitations of these active packaging materials.

Addressing these critical gaps, this study investigated how distinct polysaccharides, carboxymethylcellulose (CMC), citrus peel pectin (CPP), and potato starch (PS) influence the physicochemical properties of film-forming emulsions and, consequently, affect the retention of limonene, the main volatile terpene found in orange (*Citrus sinensis* L. Osbeck) essential oil, within edible films.

## 2. Material and Methods

### 2.1. Materials

Medium viscosity sodium carboxymethylcellulose (CMC) was purchased from Sigma-Aldrich (St. Louis, MO, USA). Low methyl ester citrus peel pectin (CPP) with a degree of methyl esterification (lower than 50) was donated by CP Kelco (GENU^®^pectin type LM102-AS-Z—Limeira, Brazil). Potato starch (PS) was purchased from C2 Alimentos (Embu das Artes, Brazil). Polyoxyethylene sorbitan monooleate (Tween 80) was purchased from Dinamica Quimica Contemporanea Ltda (Diadema, Brazil). Glycerol (analytical grade, ≥99.5% purity) was purchased from Dinâmica Química Contemporânea Ltda (Diadema, Brazil).

### 2.2. Obtaining Essential Oil from Orange Peel

Orange (*C. sinensis* L. Osbeck) samples were obtained from a local market in Campinas (São Paulo, Brazil). After juice extraction, orange peels were frozen at −18 °C, placed in polyethylene zip-lock bags (500 mL), and then ground using a MX1500 Waring^®^ laboratory blender (Waring Commercial, Stamford, CT, USA) for 60 s. The samples were stored at −18 °C prior to extraction experiments. The orange essential oil was extracted by hydrodistillation in a Clevenger apparatus for 40 min from 100 g of ground orange peel and 500 g of water. The extractions were performed in triplicate. Each extraction procedure yielded 3.7 ± 0.1 mL of orange essential oil. After extraction, the essential oil was stored at −18 °C until chemical characterization and edible film production.

### 2.3. Preparation of Film-Forming Emulsions and Edible Film Samples

The film-forming orange essential oil emulsions were prepared using 3 g biopolymer (CMC, CPP, or PS), 1 g glycerol, 0.5 g orange essential oil, 0.1 g Tween 80, and 95.4 g distilled water. The amount of 0.5 g of orange essential oil per 100 g emulsion was selected based on preliminary experiments conducted in our group, in which higher concentrations led to unstable emulsions and phase separation after casting. The suspensions with the biopolymer and glycerol were prepared 24 h before the emulsification to ensure the hydration of the materials. After that, they were stirred at 500 rpm and 80 °C for 20 min. The suspensions were cooled to 30 °C and incorporated with orange essential oil and Tween 80. Then, they were stirred at 1000 rpm for a further 10 min using a magnetic stirrer (Heidolph Scientific Products GmbH, Schwabach, Bavaria, Germany) equipped with a temperature control system.

The coarse emulsions obtained from the previous step were subjected to high-intensity ultrasound processing to emulsify the essential oil using a 13-mm ultrasound probe diameter at 19 kHz (Unique, Indaiatuba, Brazil). The probe contact height with the samples was standardized to 20 mm. The nominal power of 400 W was applied to the film-forming orange essential oil emulsions for 3 min. This nominal power delivered an acoustic power to the samples of 20 ± 1 W, as was determined by Strieder et al. [10]. All experiments were performed in triplicate.

After this step, 30 g of the film-forming emulsion was poured into glass Petri dishes (14 cm diameter) and dried at 40 °C for 24 h in a ventilated oven (Model TE-394/3, Tecnal, Piracicaba, Brazil). After drying, the films were stored in desiccators at 25 °C and approximately 50% relative humidity before further analysis. To prevent adhesion and cross-contamination, the films were individually separated by sheets of aluminum foil. The films were identified according to their biopolymer matrix as CMC (carboxymethylcellulose), CPP (citrus peel pectin), and PS (potato starch).

### 2.4. Characterization of Film-Forming Emulsions

#### 2.4.1. Microstructure

The microstructure analysis of the film-forming emulsions was carried out by optical microscopy technique using an optical microscope (Axio Scope A1, Carl Zeiss, Oberkochen, Baden-Württemberg, Germany) with 100× objective lenses and using immersion oil. The images were captured using the ZEN lite 3.11 blue edition software (Carl Zeiss, Germany).

#### 2.4.2. Droplet Size Distribution

The droplet size distribution of the film-forming emulsions was determined by the laser diffraction method using Mastersizer 2000 (Malvern Instruments Ltd., Malvern, UK). The samples were dispersed in water. The droplet size was expressed according to the volume surface mean diameter (D_3,2_) and the volume-weighted mean diameter (D_4,3_), calculated according to Equation (1) and Equation (2), respectively:

(1) $$D_{3,2}=\frac{\sum n_{i}d_{i}^{3}}{\sum n_{i}d_{i}^{2}}$$

(2) $$D_{4,3}=\frac{\sum n_{i}d_{i}^{4}}{\sum n_{i}d_{i}^{3}}$$

where $d_{i}$ is the average diameter and $n_{i}$ is the number of droplets.

#### 2.4.3. Rheological Properties

The rheological behavior of the film-forming emulsions was determined by a rheometer AR1500ex (TA Instruments, New Castle, DE, USA). A plate–plate geometry (50 mm) with a 0.5 mm gap was used. The flow curves were obtained using an up-down-up steps program. The shear rate was varied from 0.1 to 300 s^−1^. The third flow curve data were fitted to the model for power-law fluid (Equation (3)). The measurements were made in triplicate at 25 °C.

(3) $$\sigma =k{\left(\dot{\gamma }\right)}^{n}$$

where σ is the shear stress (Pa); k is the consistency index (Pa·s^n^); $\dot{\gamma }$ is the shear rate (s^−1^); and n is the flow behavior index (dimensionless).

### 2.5. Characterization of Edible Films

#### 2.5.1. Morphology

The edible films were analyzed using a high-resolution field emission gun scanning electron microscope (FEG-SEM) model Quattro SEM (Termo Fisher Scientific, Brno, Czech Republic) equipped with an energy dispersive X-ray spectrometer (EDS) model ANAX-60P-B UltraDry (Termo Fisher Scientific, Brno, Czech Republic). The samples were loaded into the SEM chamber and examined under high vacuum at an accelerating voltage of 10 kV and a 23 pA beam current.

#### 2.5.2. Functional Groups

Fourier transform infrared (FTIR) spectra of the edible films were obtained in an FTIR spectrometer (model IRPrestige-21, Shimadzu^®^, Kyoto, Japan). The mixture of 3 mg sample and 200 mg KBr powders was finely ground in an agate mortar. The mixture was compressed with a tablet press machine, forming pellets that were used in an FTIR spectrometer. The measurements were performed at room temperature, and the spectra were obtained in the range of wavenumber from 4000 to 400 cm^−1^ with a total of 10 scans at a resolution of 4 cm^−1^.

#### 2.5.3. Physical Properties

The crystallinity of the edible films was measured by the X-ray diffraction analysis on a Shimadzu XRD-6000 (Shimadzu, Tokyo, Japan) using a graphite crystal monochromator with Cu-Kα1 filter radiation of λ = 1.5406 Å at 30 kV and 30 mA. The films were analyzed in angles from 4° to 40° (2θ) with a step of 0.02° (1.2°/min)

#### 2.5.4. Thermal Stability

The thermal stability of the edible films was evaluated by thermogravimetric analysis in a TG-DTA H Shimadzu 60 machine (Shimadzu Corporation, Kyoto, Japan). The analyses were performed in a nitrogen atmosphere at a rate of 10 mL/min, with heating from 25 °C to 600 °C at a rate of 10 °C/min.

### 2.6. Essential Oil Retention

A 1.3 g piece of each edible film was dissolved in 30 mL of methanol. The mixture was then stored at 40 °C for 7 days. Afterward, the samples underwent sonication treatment for 60 min in an ultrasonic bath at 200 W and 25 kHz. An aliquot of 1.5 mL of the supernatant was filtered using a nylon membrane (0.45 µm) and injected into the Shimadzu GC-17A gas chromatograph (GC) (Shimadzu, Kyoto, Japan). The sample split ratio was 1:20. The carrier gas (Helium, 99.9% purity, White Martins, Campinas, Brazil) flowed at 1.1 mL/min. The injector and detector temperatures were set at 220 °C and 250 °C, respectively. The column was heated from 80 °C to 250 °C at a rate of 20 °C/min. The limonene was identified by comparing the retention indices of the samples and external standards. A chromatograph GC-FID (Shimadzu, CG17A, Kyoto, Japan) equipped with a capillary column of fused silica DB-5 (J&W Scientific, 30 m × 0.25 mm × 0.25 µm, Folsom, CA, USA) was used. An external standard calibration curve was used to quantify the limonene and calculate the essential oil retention [11].

### 2.7. Statistical Analysis

Analysis of variance (ANOVA) was performed using the Minitab 18^®^. The significant differences (*p*-value < 0.05) between the treatments were evaluated using Tukey’s test. The results of SEM, FTIR, DRX and TGA were analyzed descriptively.

## 3. Results and Discussion

### 3.1. Effects of Biopolymeric Matrix on Film-Forming Emulsions

The performance of edible films as carriers for essential oils depends critically on the physicochemical characteristics of the film-forming emulsions. These emulsions must not only maintain the essential oil dispersed and stabilized in the aqueous biopolymer matrix but also allow for the formation of homogeneous films that effectively entrap volatile compounds during drying. In this context, the nature of the biopolymeric matrix can influence emulsion microstructure, droplet size distribution, and rheological behavior, and these factors are directly linked to oil retention in the final film.

**Figure 1:**
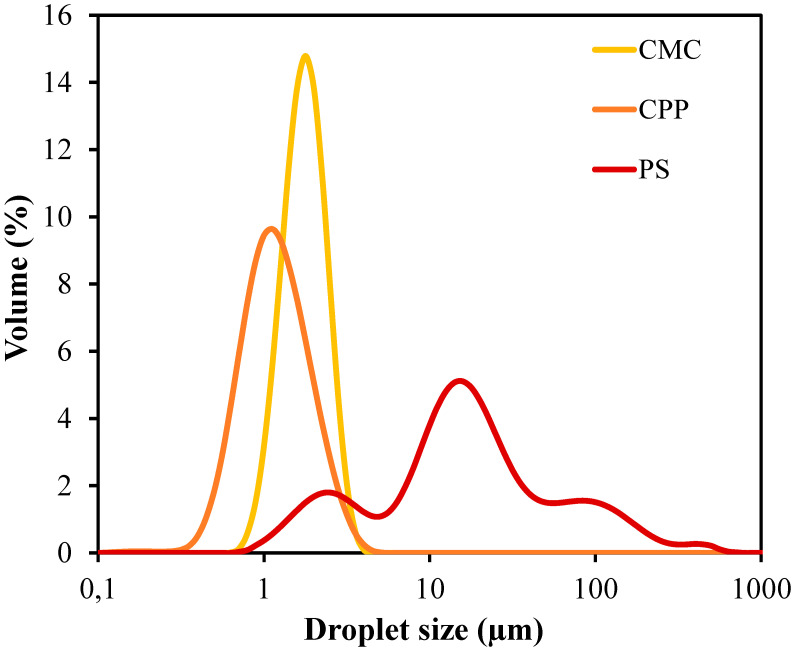
Droplet size distribution curves of film-forming emulsions produced from carboxymethylcellulose (CMC), citrus peel pectin (CPP), and potato starch (PS).

The droplet size distribution curves exhibited in Figure 1 and mean diameter values (D_3,2_ and D_4,3_) reported in Table 1 demonstrate substantial differences among the film-forming emulsions structured with CMC, CPP, and PS. The emulsions stabilized by CMC and CPP exhibited narrow and monomodal distributions, indicating uniform droplet populations, whereas the PS-based emulsion presented a broader and bimodal profile, suggesting polydispersity and potential coalescence events during or after emulsification. The distinct droplet size distribution observed for the PS-based emulsions could be attributed to the presence of ungelatinized starch granules. Potato starch typically contains granules ranging from 15 to 100 µm in size, which may have remained partially intact during emulsification, thereby contributing to the broader and bimodal particle size profile detected.

The Sauter mean diameter (D_3,2_), which reflects the surface-area-weighted mean droplet size, was significantly lower for CPP and CMC, while PS exhibited markedly larger droplets (*p*-value < 0.001). Likewise, the volume-weighted mean diameter (D_4,3_) was considerably higher for PS, in contrast to the more controlled values observed in CPP and CMC (*p*-value = 0.002). These results suggest that CMC and CPP matrices are more efficient in stabilizing oil droplets against coalescence due to their higher affinity for water, surface activity, and ability to increase the viscosity of the continuous phase. Additionally, these polysaccharides may have exhibited better compatibility or interactions with the emulsifier Tween 80, potentially enhancing emulsion stability and contributing further to droplet stabilization.

Similar trends in droplet size and stability were reported for emulsions stabilized with pectin and CMC in systems containing essential oils, supporting the key role of matrix viscosity in droplet stabilization [12,13].

**Figure 2:**
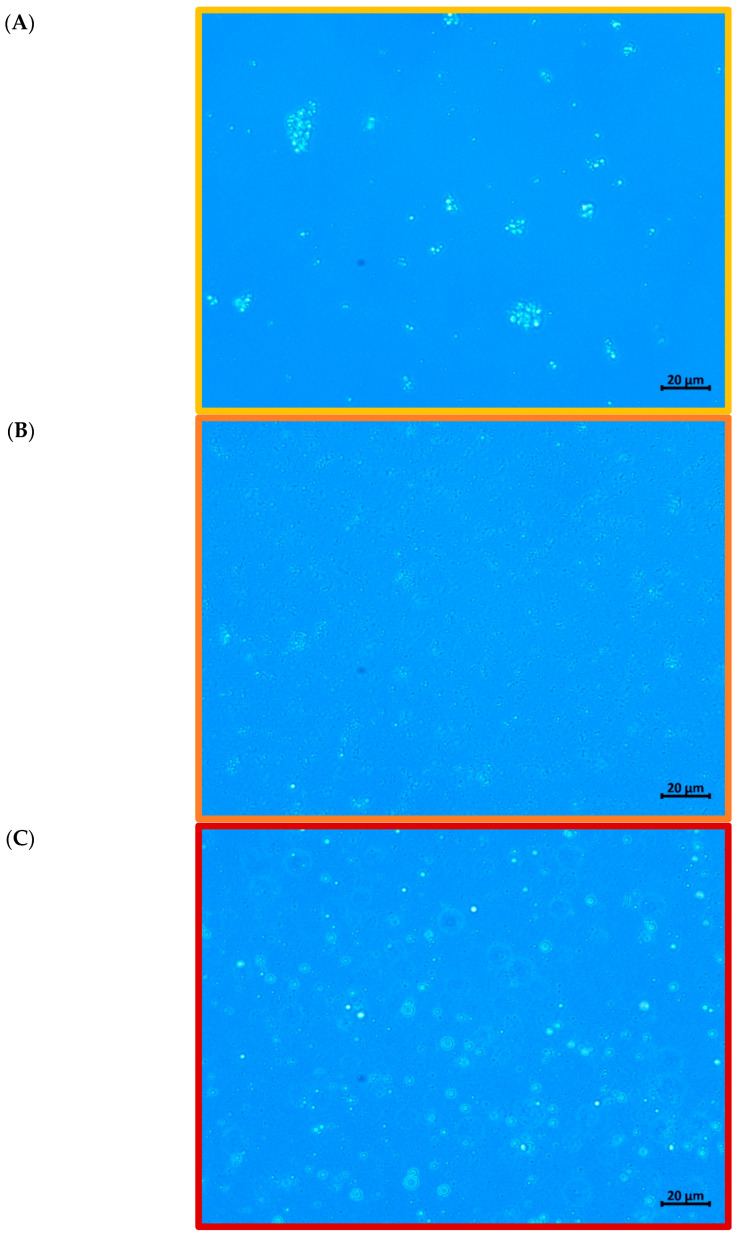
Microstructure of film-forming emulsions produced from: (**A**) carboxymethylcellulose (CMC), (**B**) citrus peel pectin (CPP), and (**C**) potato starch (PS).

Figure 2 shows optical micrographs, providing visual confirmation of the droplet size data. Film-forming emulsions containing CMC and CPP generally showed a fine and homogeneous dispersion of oil droplets, with some signs of aggregation, confirming their ability to stabilize the emulsion. In contrast, the PS-based emulsion presented larger and irregular droplets, with evident signs of flocculation and poor dispersion. The microstructural evidence suggests that potato starch lacks sufficient steric stabilizing capacity to protect the oil droplets during high-intensity ultrasound processing and subsequent handling.

This morphological instability observed in the PS film-forming emulsion may be attributed to several factors, including lower molecular flexibility, reduced solubility at the emulsification temperature, and limited interfacial adsorption capacity. Moreover, starch granules or gelatinized chains may not form strong interfacial films around oil droplets, favoring droplet coalescence and resulting in heterogeneous size distribution.

**Figure 3:**
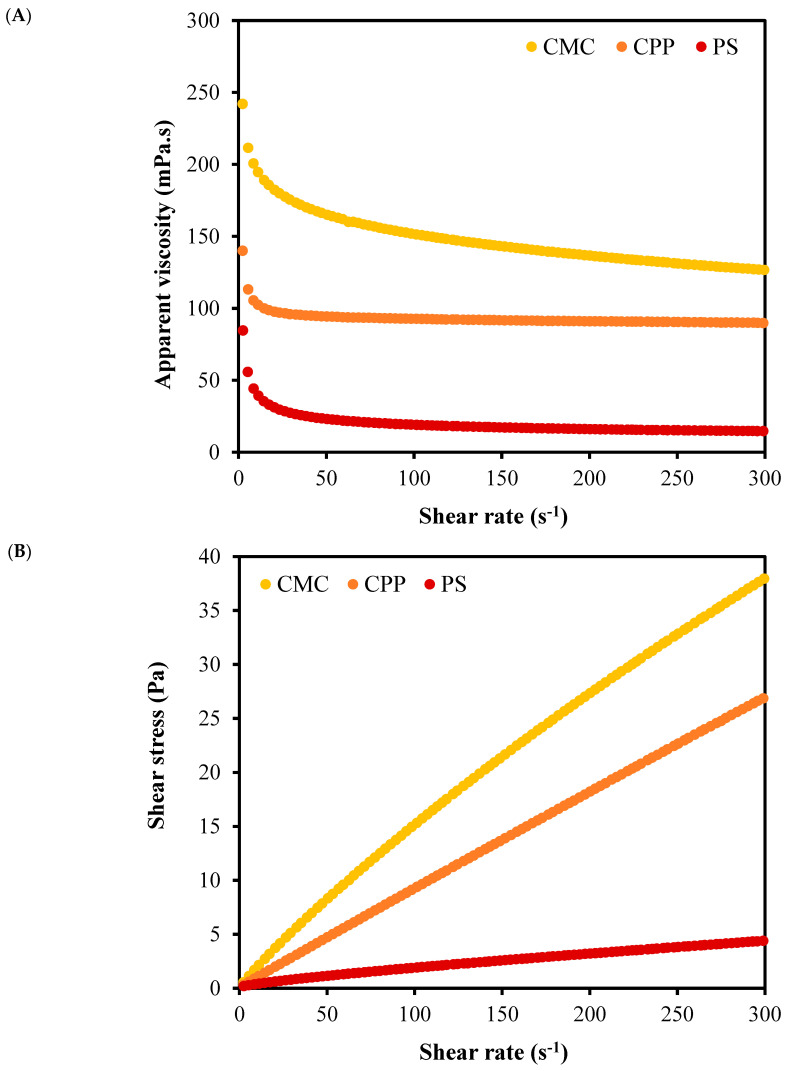
Rheological behavior of film-forming emulsions produced from carboxymethylcellulose (CMC), citrus peel pectin (CPP), and potato starch (PS): (**A**) Apparent viscosity as a function of shear rate; (**B**) shear stress as a function of shear rate.

Figure 3 and Table 1 present the rheological properties of the film-forming emulsions, providing additional insights into their structural organization and kinetic stabilization potential. All systems exhibited pseudoplastic behavior (shear-thinning), typical of polymer-based emulsions, which is favorable for film casting processes due to lower viscosity under shear and better spreadability. However, the magnitude of apparent viscosity varied considerably: CMC-based emulsions exhibited the highest viscosity, followed by CPP and PS (*p*-value < 0.001). These differences are further supported by the power-law model parameters, where the consistency index (k) was significantly higher for CMC than for CPP and PS (*p*-value < 0.001), reflecting a stronger internal structure.

Higher viscosity in CMC and CPP emulsions limits the mobility of oil droplets, increasing the resistance to coalescence and gravitational separation. This rheological profile favors the formation of more stable emulsions and, consequently, the development of uniform films with higher retention potential for volatile compounds, such as limonene. On the other hand, the low viscosity of the PS emulsion reflects a weak three-dimensional network, which is unable to restrain droplet movement, promoting phase instability and droplet growth.

### 3.2. Film Properties

#### 3.2.1. Morphology

**Figure 4:**
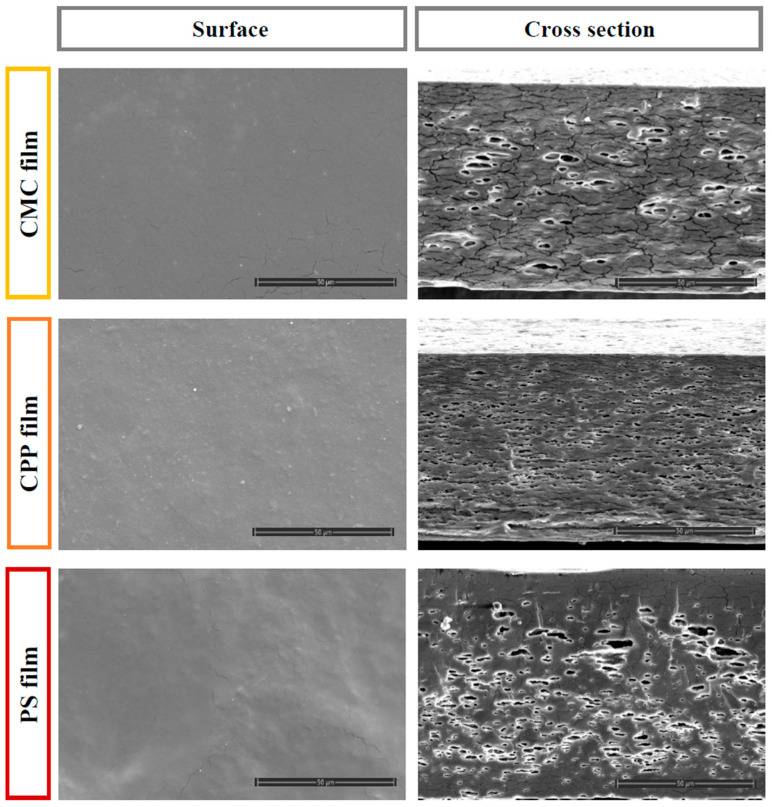
Scanning Electron Microscopy (SEM) of the surface and cross-section for each film formulation.

The microstructural characteristics of biopolymer films are influenced by interactions among their components, directly impacting their overall properties. To gain a deeper understanding of the film microstructure, including the presence of pores or cracks, scanning electron microscopy (SEM) analysis was performed. This technique enables the visualization of surface features such as cracks, bubbles, and other imperfections, thereby highlighting key properties of the films. Figure 4 presents SEM images illustrating the surface and cross-sectional views of each film at a scale of 50 µm.

The microstructural analysis of the film surfaces revealed that CPP films presented a dense distribution of pectin-derived crystalline structures without visible surface cracks, suggesting good film formation and cohesive integrity. On the other hand, films based on CMC and PS exhibited surface discontinuities, including visible cracks, particularly in the CMC formulation, where the presence of crystalline domains was slightly more pronounced. The PS-based film, while showing fewer crystalline features, presented a smoother and more homogeneous surface, which may reflect better polymer packing.

In the cross-sectional analysis, distinct differences in internal microstructure were observed among the film formulations. The CMC-based film exhibited prominent structural cracks and a small number of large, irregular pores, indicating mechanical fragility and phase separation during drying. The CPP film, on the other hand, showed a greater number of voids, though they were generally smaller and more evenly distributed, suggesting more uniform evaporation of entrapped essential oil droplets. In contrast, the PS film presented larger and deeper cavities, particularly concentrated near the upper surface, which may reflect the coalescence and migration of oil droplets during solvent removal.

Notably, the CMC film contained fewer bubbles than the other formulations, but the cracks were wider and more distinct, potentially compromising the barrier and mechanical integrity of the film. The overall structural heterogeneity observed in all samples can be primarily attributed to the volatilization and/or coalescence of hydrophobic orange essential oil droplets within the polymeric matrix during the drying step. This phenomenon is consistent with previous studies in which oil droplet instability and inadequate emulsification led to porous and heterogeneous film architectures [14].

Similar structural features have been reported in films incorporated with rosemary essential oil, particularly in pectin-based formulations combined with gelatin and chitosan. In those systems, the coalescence and aggregation of oil droplets during drying contributed to the development of heterogeneous microstructures with discontinuities across the film matrix [15]. These instabilities led to cross-sections characterized by porous, sponge-like architectures, with notable variations in pore size and spatial distribution [16]. Such configurations are known to impair structural cohesion and introduce potential failure points within the film, thereby compromising mechanical performance and barrier integrity.

#### 3.2.2. Functional Groups

**Figure 5:**
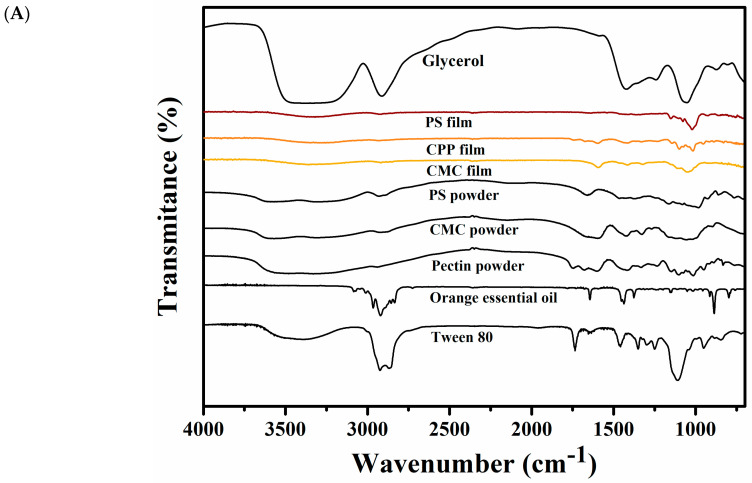

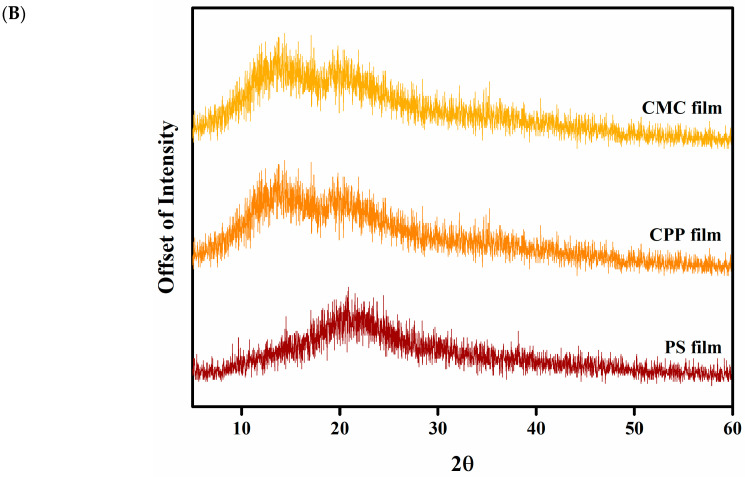
Characterization of CMC, CPP and PS-based films: (**A**) Fourier transform infrared (FTIR) spectra, and (**B**) X-ray diffraction pattern.

Figure 5A shows the FTIR spectra of CMC, CPP and PS films and all ingredients used for their formulation. The FTIR analysis identifies the main functional groups present in the polysaccharides, Tween 80, glycerol, and orange essential oil, as well as in the three formulated films.

In the FTIR spectrum of glycerol, a broad absorption band is observed between 3500 and 3200 cm^−1^, which corresponds to O–H stretching vibrations characteristic of hydroxyl groups in alcohols. A second absorption band appears in the range of 3000 to 2800 cm^−1^, associated with the C–H stretching vibrations of –CH_2_ and –CH_3_ groups. Additionally, a group of bands between approximately 1450 and 1350 cm^−1^ can be attributed to the bending (scissoring and wagging) vibrations of methylene (–CH_2_) and methyl (–CH_3_) groups. These bands reflect the aliphatic nature of the glycerol molecule and its saturated hydrocarbon backbone [17].

When analyzing the FTIR spectra of the polysaccharides (CMC, CPP, and PS), a broad band was observed between 3700 and 3400 cm^−1^, which corresponds to O–H stretching vibrations, typical of hydroxyl groups present in carbohydrate structures. In the case of starch, a peak appeared near 3000 cm^−1^, while for CMC, it was centered around 2975 cm^−1^. For pectin, this C–H stretching band was much less pronounced or nearly imperceptible. These bands are attributed to the stretching vibrations of C–H bonds in –CH and –CH_2_ groups. Additionally, a set of absorption bands starting near 1700 cm^−1^, with prominent peaks around 1600 cm^−1^ and 1450 cm^−1^, were observed in all samples. These are associated with asymmetric and symmetric stretching vibrations of carboxylate (COO^−^) functional groups, especially in CMC and CPP. A characteristic band near 990 cm^−1^ was also present, which can be attributed to C–O–C stretching in glycosidic linkages, the presence of methyl groups, or vibrations of monosubstituted alkenes, and may also reflect differences in the degree of esterification, particularly in the CPP spectrum [16,18,19].

In the FTIR spectrum of Tween 80, a broad absorption band between 3675 and 3150 cm^−1^ corresponds to the O–H stretching vibrations attributed to the polyoxyethylene groups and residual hydroxyl functionalities. Distinct peaks at 2932 cm^−1^ and 2850 cm^−1^ are assigned to the C–H stretching vibrations of methyl (–CH_3_) and methylene (–CH_2_) groups, respectively. A strong absorption band at 1730 cm^−1^ is indicative of the stretching vibration of the ester carbonyl (C=O) group, characteristic of the oleate ester present in the molecule. Finally, the peak observed around 1100 cm^−1^ corresponds to the C–O–C stretching vibrations of ether linkages in the polyoxyethylene chain [20,21].

In the FTIR spectrum of orange essential oil, a series of absorption bands are observed that reflect the presence of unsaturated hydrocarbons and minor oxygenated compounds. A set of bands between 3115 and 2725 cm^−1^ corresponds to C–H stretching vibrations, primarily from aliphatic and vinylic –CH groups. A distinct band at 1614 cm^−1^ is attributed to C=C stretching vibrations of isolated double bonds, characteristic of monoterpenes such as limonene. The peak near 1450 cm^−1^ is also related to C–H bending vibrations in –CH_2_/–CH_3_ groups and may reflect the presence of hydrocarbon chains. The band at ~890 cm^−1^ is associated with out-of-plane =C–H bending in monosubstituted alkenes, confirming the presence of terminal vinyl groups. Finally, the peak at 797 cm^−1^ may be attributed to C–H wagging or C–O stretching modes, although the latter is typically weak in hydrocarbon-rich essential oils [22].

Regarding the FTIR spectra of the edible films, all three formulations exhibited a broad absorption band around 3500 cm^−1^, corresponding to the O–H stretching vibrations. This band arises from hydroxyl groups present in both the polysaccharide matrices and glycerol and is a common feature of hydrophilic biopolymer films. In the CPP and CMC-based films, a distinct absorption band near 1660 cm^−1^ was observed, which can be associated with C=O stretching vibrations of ester groups and is indicative of the degree of esterification in pectin or residual acetylation in CMC. In the fingerprint region (below 1200 cm^−1^), all formulations displayed multiple peaks between 1160 and 820 cm^−1^, corresponding to C–O–C stretching, C–C skeletal vibrations, and ring-related modes characteristic of carbohydrate backbones. Although this region is highly informative, it is also structurally complex and often presents overlapping signals that are specific to each type of polysaccharide, making precise assignments challenging without complementary analyses [23]. Notably, some characteristic bands of orange essential oil were detected in the final film spectra. A weak but distinguishable band near 1614 cm^−1^ (C=C stretching from monoterpenes) was observed in the CMC and CPP films, suggesting the partial retention of limonene and related compounds. Additionally, the peak at ~890 cm^−1^ (out-of-plane =C–H bending) was also detected in the CPP film spectrum with greater intensity than in the PS-based film, indicating a different degree of essential oil incorporation among the matrices. These findings are consistent with the quantitative retention results and reinforce the structural evidence for oil entrapment.

#### 3.2.3. Physical Properties

X-ray diffraction (XRD) analysis was performed to investigate the crystalline and amorphous characteristics of the edible films. Crystallinity plays a pivotal role in defining the mechanical, thermal, and barrier properties of polymeric materials. While excessive or heterogeneous crystallization can introduce brittleness and structural discontinuities, the formation of well-organized crystalline domains through polymer-plasticizer or polymer-oil interactions can enhance tensile strength and film integrity [24]. The diffractograms obtained (Figure 5B) revealed predominantly amorphous profiles for all formulations, with overall crystallinity values below 10%. This low degree of crystallization suggests that the casting and emulsification techniques were effective in promoting disordered molecular arrangements, which are typically desirable in films intended for flexible packaging applications. All film formulations exhibited a broad peak centered near 2θ ≈ 21°, which is commonly associated with amorphous polysaccharide structures [25].

Among the samples, the CMC-based film presented a broad halo with a subtle crystalline peak at 2θ = 20.71°, corresponding to a crystallinity index of approximately 4.57%, indicating limited molecular ordering. The pectin film displayed a more complex profile, with a low-intensity peak at 2θ = 13.63°, characteristic of pectin-based films and often linked to localized microstructural rearrangements or agglomerates. Additionally, a secondary peak at 2θ = 19.25° was observed, resulting in the highest crystallinity among the formulations (~9.97%), which may reflect interactions between pectin chains and the essential oil or plasticizer. The potato starch (PS) film exhibited a primary peak at 2θ = 20.23°, with an intermediate crystallinity level of 5.80%. This pattern suggests partial retrogradation or reorganization of starch molecules during film formation. However, the broadness of the peak still indicates a mostly amorphous matrix. These results are consistent with previous studies on polysaccharide-based films containing essential oils [12,26].

The XRD data support the conclusion that the film matrices are predominantly amorphous, favoring the uniform dispersion and possible entrapment of hydrophobic components such as limonene. The slight differences in crystallinity among formulations, with CPP films showing the highest degree of structural organization, which could contribute to their superior oil retention performance.

#### 3.2.4. Thermal Stability

Thermogravimetric analysis (TGA) was performed to evaluate the thermal stability and degradation behavior of biopolymer-based edible films. This technique provides valuable insights into the moisture-binding capacity, decomposition stages, and intermolecular interactions within the polymeric matrix, all of which influence the film’s structural integrity and performance during storage or thermal processing [27].

**Figure 6:**
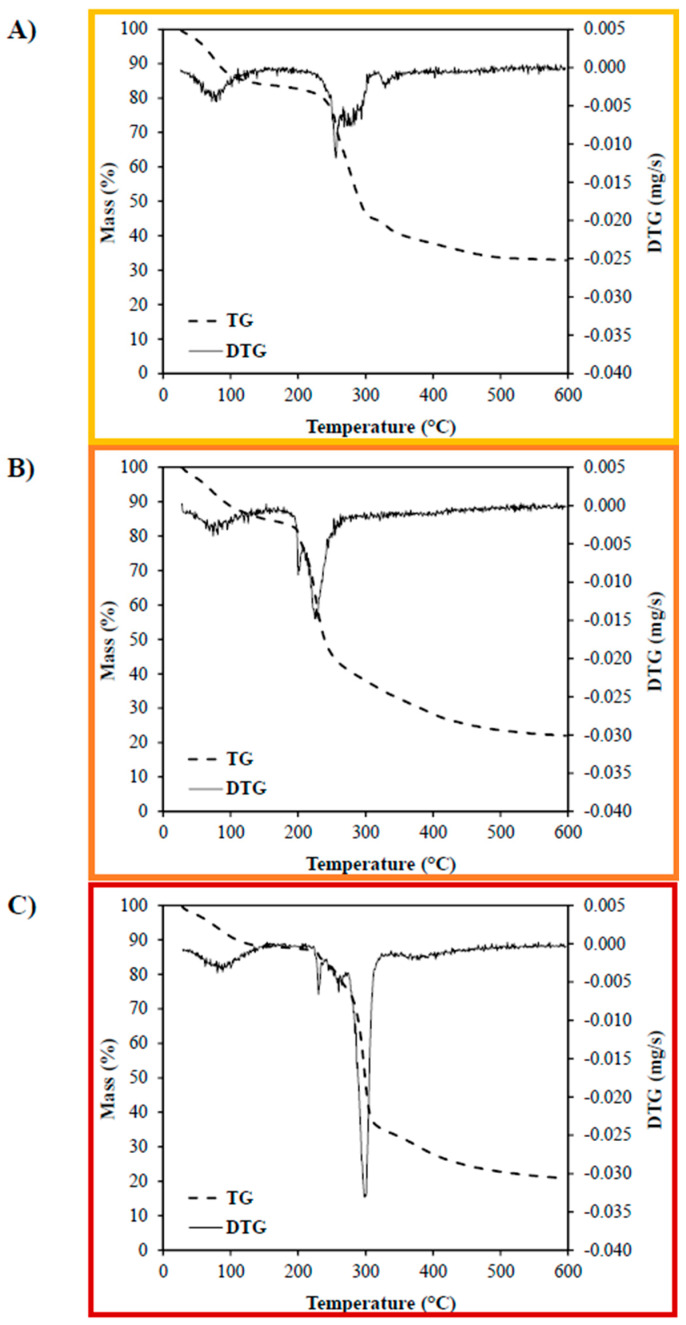
Thermogravimetric analysis of edible films produced from (**A**) carboxymethylcellulose (CMC), (**B**) citrus peel pectin (CPP), and (**C**) potato starch (PS).

The thermogravimetric profiles (Figure 6) revealed a three-step degradation pattern for all film formulations. The first mass loss stage (35–90 °C) was associated with the evaporation of free and crystalline water, with weight losses of approximately 12% for CMC, 10% for CPP, and 8% for PS films. These differences reflect the hygroscopic nature of each polysaccharide, with CMC exhibiting the highest affinity for moisture. The second stage (90–150 °C) is associated with the evaporation of bound water, often retained via hydrogen bonding within the polymer network. Mass losses during this stage were relatively similar across formulations, 4% (CMC), 5% (CPP), and 4% (PS), suggesting comparable levels of molecular water entrapment. The third stage, corresponding to the thermal degradation of the polymer matrix, showed significant differences among the formulations. The main decomposition temperatures were 258 °C for CMC, 234 °C for CPP, and 299 °C for PS films. The degradation peaks for CPP and PS films were consistent with those reported in the literature for pectin and native starch matrices, respectively, indicating that the incorporation of essential oil and plasticizer did not significantly alter their primary decomposition pathways [28,29].

Interestingly, the CMC film displayed four degradation events, including a distinct peak at 330 °C, not observed in the other formulations. This additional event may reflect stronger molecular interactions between the CMC chains and the essential oil, likely mediated by hydrogen bonding between the polar groups of limonene and the carboxymethyl groups of the polymer. However, the maximum degradation temperature of the CMC film (258 °C) was slightly lower than that of pure CMC (typically ~280 °C), suggesting that the presence of essential oil may also disrupt some original interchain interactions, leading to rearrangement of molecular packing and altered thermal behavior [30,31].

Overall, the TGA results demonstrate that while all films exhibit good thermal stability for food packaging applications, CMC films appear to interact more strongly with the essential oil, as evidenced by the altered degradation profile. In contrast, the PS and CPP matrices maintained more conventional degradation patterns, with CPP showing slightly lower thermal resistance, possibly due to its higher porosity and lower structural compactness observed in the microstructural analyses.

### 3.3. Limonene Retention

**Figure 7:**
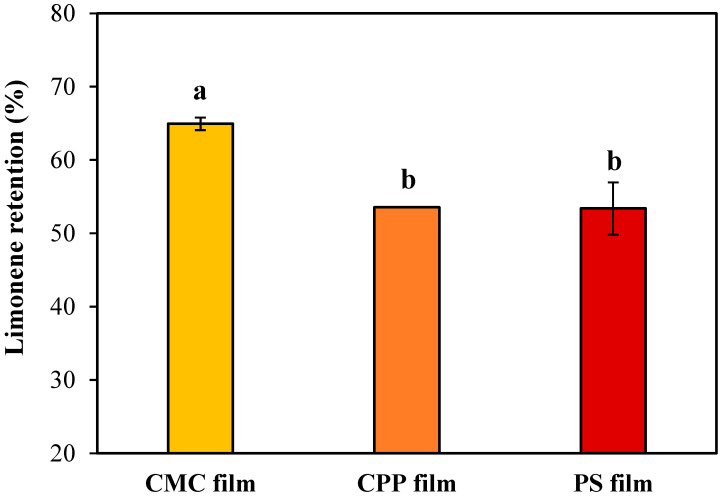
Effect of a biopolymeric matrix of the edible film on limonene retention. Mean values labeled with different letters are significantly different according to Tukey’s multiple comparison test (*p*-value < 0.05).

The efficiency of essential oil retention in edible films is a critical factor in determining their functionality in active food packaging systems. In this study, limonene retention was evaluated as a marker of how effectively each polysaccharide-based matrix, CMC, CPP, and PS, was able to entrap and preserve volatile bioactive compounds during film formation and drying by casting. As shown in Figure 7, the CMC-based films retained the highest amount of limonene, while CPP and PS-based films showed similar and significantly lower retention levels (*p*-value = 0.019).

CMC-based films exhibited the highest limonene retention (approximately 65%). Although this retention level is moderate, it represents a promising outcome for hydrophilic polysaccharide films encapsulating volatile hydrophobic compounds, given the inherent challenges associated with the volatilization of such molecules during drying. The superior retention observed for the CMC films may be primarily explained by their emulsion characteristics, particularly the notably higher viscosity (0.15 ± 0.01 Pa·s), which potentially restricted droplet mobility and minimized droplet coalescence during drying. Additionally, the cross-sectional SEM images indicated that the internal structures of the CMC films featured large, irregular cavities formed by the volatilization of oil droplets. Despite these cavities, the high viscosity of the CMC emulsion likely reduced limonene migration and loss, thereby enhancing overall retention efficiency.

On the other hand, PS-based films, despite exhibiting similar cross-sectional morphologies to the CMC films, with comparable cavity sizes and structures, had significantly lower limonene retention (~53%). This observation underscores that structural similarity alone cannot guarantee similar retention levels. Other critical factors, including the larger oil droplet sizes (D_3,2_ = 7.01 ± 0.05 µm, D_4,3_ = 35 ± 5 µm), lower viscosity (0.019 ± 0.002 Pa·s), and potentially weaker interactions between starch and limonene, likely contributed to greater droplet coalescence, oil migration to the film surface, and increased volatilization during the drying process.

CPP-based films presented limonene retention statistically similar to the PS films, despite having the smallest droplet sizes (D_3,2_ = 1.0 ± 0.1 µm, D_4,3_ = 1.2 ± 0.1 µm) and intermediate viscosity (0.093 ± 0.005 Pa·s). The higher porosity observed in the cross-sectional SEM images, characterized by numerous small and uniformly distributed pores, may have facilitated the diffusion and escape of limonene during drying. Consequently, although favorable emulsion characteristics (small droplet size and moderate viscosity) were achieved, these were insufficient to prevent significant limonene losses.

It is also important to highlight that, in this study, the emulsifying action was provided by Tween 80. Therefore, differences in droplet stabilization and retention performance among the films should be attributed primarily to the viscosity of the continuous phase (governed by the polysaccharides) and the physical entrapment of limonene within the structural network formed during drying rather than the direct emulsifying properties of the biopolymers.

The maximum retention level (~65%) achieved by CMC-based films, although promising, indicates substantial scope for further optimization. While polysaccharide matrices can serve as carriers of volatile compounds, their inherent hydrophilic nature poses intrinsic limitations. Strategies such as employing multilayer film systems, increasing drying rates, combining polysaccharides with hydrophobic or amphiphilic biopolymers, or optimizing emulsifier type and concentration could potentially enhance limonene retention and, thus, the functional efficacy of active packaging.

## 4. Conclusions

The results revealed that the type of biopolymer significantly influenced the limonene retention capacity, driven primarily by differences in emulsion characteristics, matrix viscosity, and film structure. CMC-based films demonstrated the highest limonene retention (~65%), attributed mainly to their notably higher emulsion viscosity, which restricted droplet mobility and limited volatilization losses during drying by casting. On the other hand, PS-based films showed significantly lower retention despite presenting similar internal morphologies with comparable cavity structures. This difference highlights the importance of droplet size, emulsion stability, and viscosity in volatile retention. CPP films exhibited intermediate characteristics, with small droplet sizes and moderate viscosity but highly porous structures, resulting in limonene retention statistically similar to PS films. These findings indicate that while polysaccharide matrices, especially CMC, can effectively encapsulate hydrophobic volatile compounds, inherent limitations remain. Future work should explore combinations of hydrophilic and hydrophobic biopolymers, fast-drying technologies, or nanoemulsification to further improve encapsulation efficiency and broaden the industrial applicability of these systems.

## Figures and Tables

**Table 1:** Mean diameters and rheological properties of film-forming emulsion produced from carboxymethylcellulose (CMC), citrus peel pectin (CPP), and potato starch (PS).

Film-Forming Emulsion	D_3,2_ (µm)	D_4,3_ (µm)	Apparent Viscosity at 101 s^−1^ (Pa.s)	k (Pa.s^n^)	n	R^2^
CMC	1.57 ± 0.01 ^b^	1.71 ± 0.02 ^b^	0.15 ± 0.01 ^a^	0.274 ± 0.004 ^a^	0.87 ± 0.01 ^b^	0.999
CPP	1.0 ± 0.1 ^c^	1.2 ± 0.1 ^b^	0.093 ± 0.005 ^b^	0.12 ± 0.01 ^b^	0.952 ± 0.002 ^a^	0.999
PS	7.01 ± 0.05 ^a^	35 ± 5 ^a^	0.019 ± 0.002 ^c^	0.082 ± 0.004 ^c^	0.69 ± 0.01 ^c^	0.998

D_3,2_—Sauter mean diameter; D_4,3_—De Brouckere mean diameter; k—consistency index; n—flow behavior index. Different letters in the same column indicate a significant difference (*p*-value < 0.05) by the Tukey test.

## Data Availability

The original contributions presented in this study are included in the article. Further inquiries can be directed to the corresponding author.
